# Organic Certification is Not Enough: The Case of the Methoxydecane Frankincense

**DOI:** 10.3390/plants8040088

**Published:** 2019-04-04

**Authors:** Stephen Johnson, Anjanette DeCarlo, Prabodh Satyal, Noura S. Dosoky, Aaron Sorensen, William N. Setzer

**Affiliations:** 1 Aromatic Plant Research Center, 230 N 1200 E, Suite 100, Lehi, UT 84043, USA; sjohnson@aromaticplant.org (S.J.); psatyal@aromaticplant.org (P.S.); ndosoky@aromaticplant.org (N.S.D.); asorensen@aromaticplant.org (A.S.); 2 Department of Chemistry, University of Alabama in Huntsville, Huntsville, AL 35899, USA

**Keywords:** methoxydecane, decyl methyl ether, octyl methyl ether, frankincense, *Boswellia carteri*, *Boswellia sacra*, *Boswellia occulta*, olibanum, organic certification, non-timber forest products

## Abstract

Frankincense, the oleo-gum-resin of *Boswellia* trees, has been an important religious and medicinal element for thousands of years, and today is used extensively for essential oils. One of the most popular frankincense species is *Boswellia sacra* Flueck. (syn. *Boswellia carteri* Birdw.) from Somalia and Somaliland. Recent increases in demand have led to many areas being overharvested, emphasizing the need for incentives and monitoring for sustainable harvesting, such as certification schemes. Concurrently, a new chemical component, called methoxydecane, has emerged in oils claimed to be *B. carteri*, suggesting the possibility of a chemical marker of overharvesting or other stress that could aid in monitoring. To find the source of this new chemical component, we sampled resin directly from trees in areas producing the new methoxydecane chemotype. This revealed that methoxydecane comes not from *Boswellia carteri*, but from a newly described frankincense species, *Boswellia occulta*. The presence of *Boswellia occulta* oil in essential oil sold as pure *B. carteri*, including certified organic oil, emphasizes the current lack of traceability in the supply chain and the ineffectiveness of organic certification to secure purity and sustainable harvesting in wildcrafted species.

## 1. Introduction

Frankincense is an aromatic oleo-gum-resin produced by members of *Boswellia* (Burseraceae: Sapindales), a genus of dry paleotropical trees generally characterized by papery, exfoliating bark, compound leaves, and the production of aromatic resin [1]. Ecologically, the resins are thought to be important as deterrents to herbivory, infection, attack by boring beetles, and other forms of damage [2,3]. As a product, it has been traded and utilized in traditional medical systems for up to 5000 years, making it one of the oldest commodities in the world [2,4,5]. There are about 20 members of the genus, distributed across Africa, Arabia, and South Asia; almost all are used locally, but only a few produce resins that are traded in significant volumes on the international market: *Boswellia papyrifera* Hochst., *B. serrata* Roxb. ex Colebr., *B. sacra* Flueck. (syn. *B. carteri* Birdw.), and *B. frereana* Birdw. The resins are used as incense and medicine, and are frequently distilled into essential oils, which are popular in perfumery and aromatherapy.

*Boswellia sacra* syn. *B. carteri* is one of the most popular frankincense essential oils for both aromatherapeutic applications and in perfumes. The status of *B. carteri* as a separate species is questionable, typically being considered botanically as being part of a single, highly variable species, *B. sacra* [6]. However, the two show differences in morphology, growth form, resin chemistry, and geography; the Arabian populations are termed *B. sacra* while the African populations are termed *B. carteri* [1,6,7] (personal observations). Additionally, much of the scientific literature, including the phytochemical literature, refers to *B. carteri* as distinct from *B. sacra*, and it is recognized commercially as a separate entity [8,9,10,11]. For these reasons, we here refer to the African populations of *B. sacra* syn. *B. carteri* simply as *Boswellia carteri*. *Boswellia carteri* is native to Somaliland and the Puntland state of Somalia. It grows on limestone and volcanic rock (as well as occasionally on soil) from sea level up to 1500 m in elevation [6,12] (personal observations).

The trees are harvested for frankincense across their range by making small cuts into the cambium and scraping off the exuded resin. Proper harvesting, according to traditional best practices, does not unnecessarily harm the tree or decrease its lifespan; overharvesting, by contrast, increases mortality and significantly reduces reproductive output [13,14,15,16] (personal observations). In Somaliland, harvesting intensity has increased markedly over the past decade due to increasing market demand, resulting in trees in many areas being tapped far beyond the traditional limits (personal observations). Although quantitative data have been hard to obtain, qualitative observations and interviews with key informants suggest that overharvesting is the most significant threat to the frankincense trees’ survival in Somaliland [17,18] (personal observations). 

As a possible solution, certification schemes have been suggested as a way to ensure sustainable harvesting and prevent exploitation of harvesting communities [19,20,21,22]. A number of certification standards, most notably FairWild, are specifically designed for the harvesting of wild plants, and have been implemented successfully elsewhere; however, they are data-intensive, requiring information such as population inventories and landscape assessments that can be difficult to gather in Somaliland. Organic certification offers a less intensive option that also demands greater traceability and provides economic benefits; at least four companies are certified to produce organic resins in Somaliland/Somalia to United States Department of Agriculture National Organic Program (USDA NOP) standards [23]. By requiring a greater degree of traceability, organic certification may help ensure that trees are sustainably harvested in the areas where the resins are sourced.

In addition to certification schemes, the presence of a chemical marker of overharvesting or tree stress would provide a powerful tool for monitoring harvesting practices [24]. The majority of resins are distilled for essential oil, which is routinely tested by purchasing companies for purity, thereby providing an opportunity to routinely screen resins for the presence of marker compounds. Concurrently with the increase in overharvesting, a new, unusual chemotype dominated by methoxydecane and methoxyoctane was identified from frankincense coming from Somaliland [25,26]. The resin is visually similar to *B. carteri* and *B. frereana* resin, but the essential oil is composed primarily of 1-methoxydecane (decyl methyl ether, 30–60%) and 1-methoxyoctane (octyl methyl ether, 5–15%), with only minor levels of the usual terpenes; the resin yields approximately 1–3% essential oil [25,26]. This contrasts markedly with the other chemotypes identified for *B. carteri* or any other known *Boswellia*, which are all dominated by terpenes, fatty alcohols, or fatty esters, particularly α-pinene, α-thujene, limonene, myrcene, sabinene, octyl acetate, and octanol; *B. carteri* resins also typically have essential oil yields ranging from 4% to 8% [7,8,9,10,11,25,27,28,29,30,31,32,33,34,35,36,37].

The reasons for the unusual chemotype and only recent appearance have not been established, but one hypothesis is that it is a chemical response to prolonged overharvesting, representing a chemical marker of overharvested trees. Other explanations include that rising demand and local prices for resin have motivated tapping of previously undisturbed areas, or that it is produced by *B. carteri* trees during drought. A key implication of all of these hypotheses is that the frankincense trees in Somaliland are experiencing heightened stress, either from intensifying/expanding resin harvesting or from significant environmental stress. Both certification of harvested trees and chemical stress markers may therefore be important tools to encourage sustainable harvesting and protection of harvested trees. In this paper, we aim to identify the source trees producing the methoxydecane chemotype, in order to better understand its cause and the implications of its emergence for sustainable forest management.

## 2. Results

### 2.1. Botanical Identification of the Methoxydecane Tree

**Figure 1:**
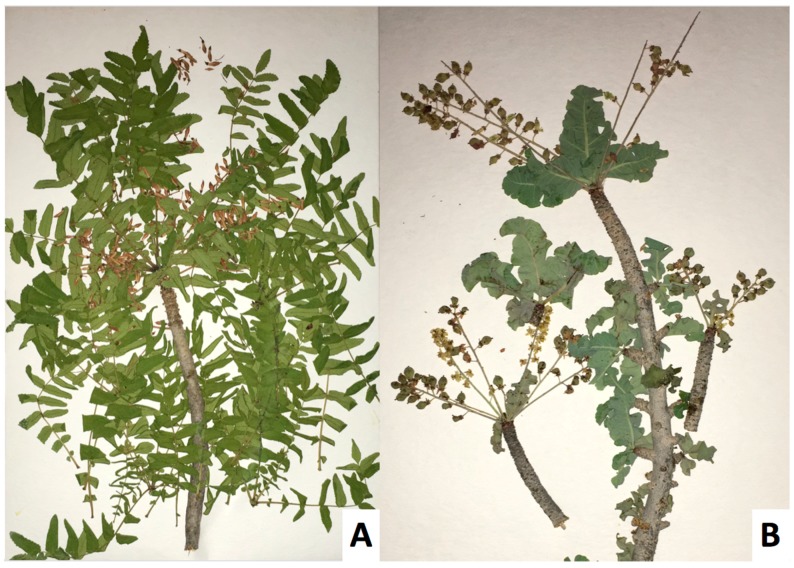
Specimens of *Boswellia carteri* (**A**) and the methoxydecane-producing tree (**B**) *Boswellia occulta*.

**Figure 2:**
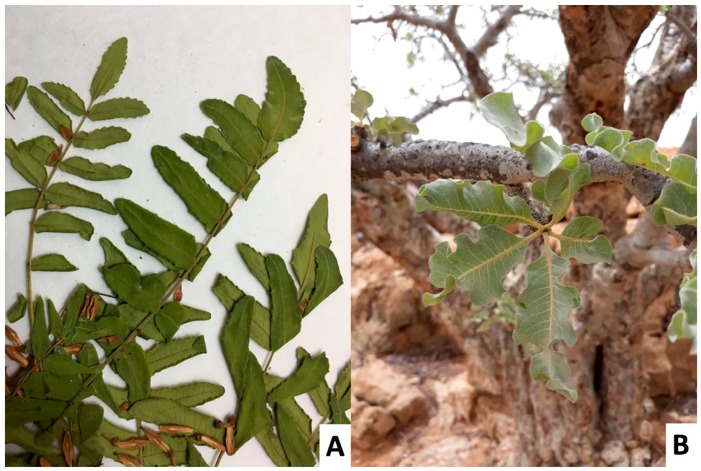
*Boswellia carteri* compound leaves (**A**) and *Boswellia occulta* simple leaves (**B**).

**Figure 3:**
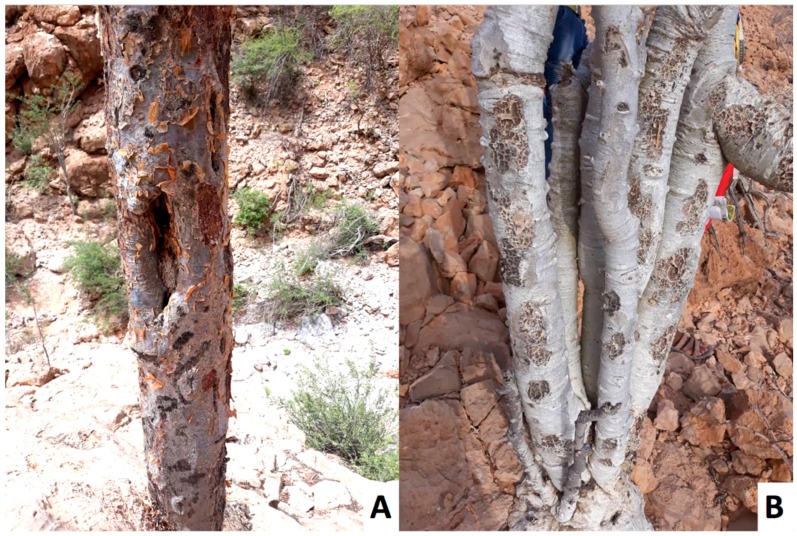
*Boswellia carteri* (**A**) and *Boswellia occulta* (**B**) trunks, showing exfoliating bark on the former but not the latter.

**Figure 4:**
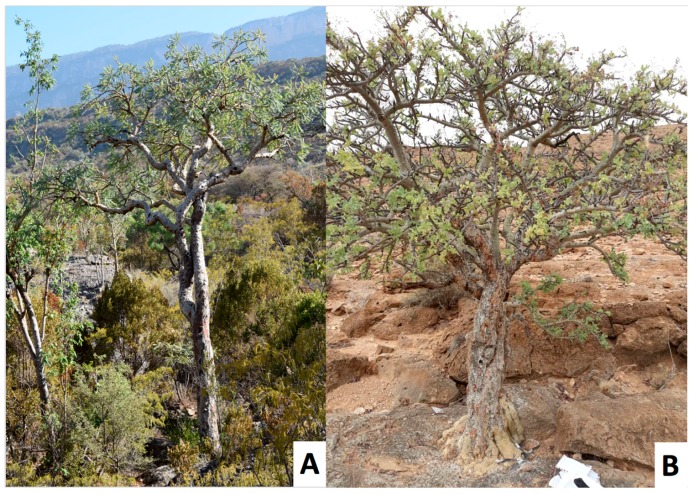
*Boswellia carteri* (**A**) and *Boswellia occulta* (**B**) in situ.

The methoxydecane tree was identified as a new species of *Boswellia*, named *Boswellia occulta* Thulin, DeCarlo & S. P. Johnson, and described in [38]. It shows a highly distinct morphology from *Boswellia carteri*: *B. occulta* has glabrous, compound leaves and smooth gray bark, in contrast to the pubescent, compound leaves and exfoliating bark of *B. carteri* (see Figure 1, Figure 2, Figure 3 and Figure 4).

### 2.2. Chemical Composition of Resin Samples

Essential oil from the oleo-gum-resin of the methoxydecane frankincense was obtained by hydrodistillation in amounts ranging from 2.68% to 5.45%. All samples were dominated by decyl methyl ether (26.6–47.9%), with smaller amounts of octyl methyl ether (3.6–9.2%) (Table 1). Serratol was also prominent in most samples (2.7–31.8%). Other components included sabinene, incensole, germacrene D, verticilla-4(20),7,11-triene, and an unidentified sesquiterpene alcohol (Table 1).

### 2.3. Commercial Essential Oil Results

The presence of methoxydecane was detected in 9 out of the 13 essential oil samples tested, in amounts ranging from 0.1% to 17.7% (median = 7.8%). It was also detected in 4 out of the 5 certified organic essential oils, in amounts ranging from 7.8% to 12.11% (median = 9.2%) (see Table 2).

## 3. Discussion

The purpose of this study was to identify the reason for the emergence of a new chemical component, methoxydecane, in essential oils of *Boswellia carteri* coming from Somaliland. Our results show that the presence of methoxydecane is not due to either climatic or anthropogenic stress, as previously hypothesized, but rather to the mixing of resin from two different species, *Boswellia carteri* and *Boswellia occulta*. Although it is possible that both species produce this chemotype, it is unlikely given that methoxyalkanes are extremely rare as natural products, only being reported in a handful of arthropods, possibly microbes, and never, to our knowledge, in plants [39,40,41,42,43]. Furthermore, methoxyalkanes have never appeared in resin samples from confirmed *B. carteri* trees [17]. Therefore, we conclude that methoxyalkanes, and especially methoxydecane, are the hallmark of *B. occulta* rather than of overharvested trees.

**Figure 5:**
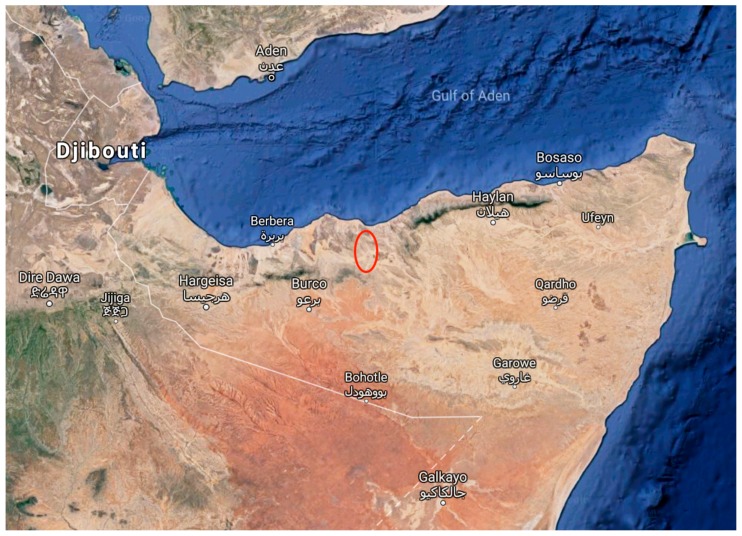
Potential range of *Boswellia occulta* based on reports and sampling locations.

Despite the obvious morphological differences, Somali harvesters have considered *Boswellia carteri* and *Boswellia occulta* to be the same species, referring to them both as “Mohor,” while drawing a distinction between these two species and another commonly harvested frankincense, *Boswellia frereana* (“Yegcar”). The harvesters do distinguish multiple types of *B. carteri,* based on growth form, resin characteristics, and substrate (Mohor cad, Mohor lab, Mohor dadbeed; *B. occulta* is called Mohor madow), but they refer to the resin of both *B. carteri* and *B. occulta* as “beeyo,” while the resin of *B. frereana* is called “maydi” ([6,12], personal communications with harvesters). While locally common, *Boswellia occulta* appears to inhabit a geographically limited range in the Sanaag region of Somaliland, primarily around the towns of Ceel Dibir and Bildhalaay in Western Sanaag (personal communications with harvesters and exporters, Figure 5). This may help explain why it was not previously identified; still, its anonymity until now, despite its prevalence in frankincense essential oils, indicates that companies selling the resin or essential oil likely have limited knowledge of the source trees of these resins.

Although methoxydecane has only shown up in essential oils in significant quantities within the last few years, harvesters claim that there is a long history of the resin’s use, primarily as incense sold into the Arabian market. Its sudden appearance in essential oils therefore may be due to increasing demand for resin from essential oil companies; the war in Yemen closing a key traditional trading route for incense to Arabia may also be a contributing factor (personal communications with harvesters and exporters).

Certification of non-timber forest products has been suggested as a way to increase product value, improve harvesting sustainability, provide greater social benefits, and improve market access [19,20,21,22]. However, certification schemes in wild products are often handicapped by limited ecological knowledge of the target species and its harvesting [22]. This is particularly true in places like Somaliland and Somalia, where access to the harvesting areas and research effort has been limited [18]. Still, at least four companies are certified to produce organic frankincense resins in Somaliland and Somalia to USDA NOP standards [23]. Certification is financially advantageous for companies, as *Boswellia carteri* essential oils that are certified organic tend to sell for higher prices than non-certified essential oils, although artificial chemicals are not applied to frankincense trees anywhere in Somaliland or Somalia, to our knowledge (personal communications with harvesters, personal observations).

In this study, four out of five of the essential oils claiming to be certified organic *Boswellia carteri* tested positive for the presence of methoxydecane, containing on average even more methoxydecane than the conventional oils. This indicates that organic certification, while useful in preventing artificial chemical contamination, is not sufficient to guarantee purity in wild-harvested products. There are several reasons why this contamination is possible even in certified resins. Certifiers may not have sufficient knowledge to accurately identify the species they are supposed to certify, or they may only visit a small subset of the total area that they certify. Alternatively, resins coming from non-certified areas may be passed off as certified material. Regardless of the reasons, the pervasiveness of methoxydecane in commercial essential oils being sold as pure *B. carteri* essential oil demonstrates the ineffectiveness of organic certification alone at preventing biological contamination. As a corollary, this also indicates that organic certification is unlikely to ensure sustainable harvesting practices, as the certification apparently is not able to accurately identify source trees. Therefore, sustainability efforts should concentrate on robust traceability and verification frameworks and incentives for sustainable harvesting rather than only organic certification.

## 4. Materials and Methods 

### 4.1. Sample Collection

Areas producing methoxydecane resins were identified by a major resin exporter in Somaliland and confirmed via commercial samples from each area; specific villages were identified by local harvesters. Samples of oleo-gum-resin were collected (March–July 2018) by our field team directly from 12 individual trees being tapped by local harvesters and identified by these harvesters as *Boswellia carteri*. The resins were sealed in plastic bags and shipped to The Aromatic Plant Research Center in Utah, USA, for analysis. Additionally, the team took photographs of each tree (canopy, leaves, and bark), as well as a voucher specimen which was deposited into the University of Hargeisa Herbarium (HARG No. 000189).

In addition, 13 oil samples from 12 common essential oil brands, all of which claim to be pure *Boswellia carteri* essential oil, were purchased and screened for the presence of methoxydecane, to identify the extent of its presence in the market. Of these 13 oil samples, 5 are claimed to be organic certified.

### 4.2. Sample Identification

The voucher specimen and in situ photographs were examined and identified by Dr. Mats Thulin, a botanical taxonomist (Systematic Biology, Department of Organismal Biology, EBC, Uppsala University, Uppsala, Sweden) with particular expertise in *Boswellia*.

### 4.3. Hydrodistillation

Hydrodistillations of the oleo-gum-resin samples of *B. occulta* were carried out using an all-glass Clevenger apparatus as previously described [25].

### 4.4. Gas Chromatographic–Mass Spectral Analysis

The *B. occulta* oleo-gum-resins and commercial *Boswellia carteri* essential oils were analyzed by GC-MS with a Shimadzu GCMS-QP2010 Ultra with ZB-5 capillary column as previously described [25]. Identification of the chemical components was carried out by comparison of the retention indices determined with respect to a homologous series of normal alkanes and our comparison of their mass spectra with those reported in the literature [44] and our own in-house library [45].

## 5. Conclusions

Methoxydecane in frankincense essential oil comes from a new species, *Boswellia occulta* Thulin, DeCarlo & S. P. Johnson, rather than being a chemotype of *Boswellia carteri*, as previously thought. The new species is highly morphologically distinct from *B. carteri*, and likely evaded detection until now due to a lack of traceability and source verification in most frankincense supply chains. Methoxydecane has been detected in many commercial essential oils being sold as pure *Boswellia carteri*, including in certified organic essential oils, indicating both the need for improved supply chain transparency and the ineffectiveness of organic certification in ensuring purity and sustainable harvesting in these wild-harvested species.

## Figures and Tables

**Table 1:** Major components (average percentages and ranges) found in *Boswellia carteri* and *Boswellia occulta* oleo-gum-resin essential oils.

Compound	*Boswellia carteri* ^a^				*Boswellia* *occulta*
	Chemotype Ia	Chemotype Ib	Chemotype Ic	Chemotype II	
α-Pinene	27.6 (14.2–39.8)	45.3 (43.2–50.2)	26.8 (24.1–32.3)	8.1 (2.3–14.8)	0.5 (0.1–2.4)
Limonene	7.9 (0.8–14.9)	11.5 (5.9–18.6)	32.5 (28.0–44.8)	2.0 (1.0–4.8)	0.1 (trace–0.1)
α-Thujene	5.0 (trace–14.0)	4.8 (0.8–11.7)	2.1 (0.1–3.6)	40.6 (32.9–50.6)	0.3 (0.1–0.6)
Sabinene	8.1 (1.4–25.7)	3.7 (2.3–7.0)	3.8 (0.4–4.9)	7.4 (5.6–10.9)	3.5 (0.1–8.3)
Myrcene	10.9 (0.4–25.7)	3.5 (2.3–12.1)	2.9 (0.0–4.2)	0.8 (0.0–2.9)	0.1 (0.0–0.1)
*p*-Cymene	5.3 (0.7–11.8)	2.7 (1.7–5.0)	3.2 (1.7–4.1)	13.0 (4.3–19.7)	0.3 (0.1–0.8)
1-Methoxydecane	0.0	0.0	0.0	0.0	35.1 (26.6–47.9)
Serratol	0.1 (0.0–0.4)	1.0 (0.0–5.9)	0.2 (0.0–0.6)	0.0	15.1 (2.7–31.8)
Unidentified guaiol ^b^	0.0	0.0	0.0	0.0	9.7 (1.2–15.1)
1-Methoxyoctane	0.0	0.0	0.0	0.0	6.4 (3.6–9.2)

^a^*Boswellia carteri* chemotypes [25]: Ia (α-pinene/myrcene/sabinene/limonene), Ib (α-pinene/limonene), Ic (limonene/α-pinene), II (α-thujene/*p*-cymene). ^b^ RI: 1607. MS(EI): 222(3%), 204(57%), 189(73%), 175(13%), 161(100%), 147(25%), 133(33%), 119(62%), 107(61%), 105(87%), 95(26%), 93(53%), 91(41%), 81(39%), 79(36%), 69(22%), 67(19%), 59(65%), 55(32%), 43(38%), 41(36%).

**Table 2:** Chemical components found in commercial samples of frankincense oils.

Compound	Non-Certified Commercial Samples								Certified “Organic” Samples				
	1	2	3	4	5	6	7	8	9	10	11	12	13
α-Pinene	26.6	31.5	32.6	24.4	40.4	39.5	39.9	34.8	42.3	47.5	37.3	35.1	35.7
Limonene	15.7	25.7	5.7	5.1	6.3	9.7	19.2	9.9	4.5	4.5	5.8	8.1	17.5
α-Thujene	11.2	7.3	1.6	1.2	11.9	10.6	6.1	7.2	1.4	2.2	1.3	1.8	6.2
Sabinene	5.8	4.1	5.4	6.2	1.3	4.2	6.0	6.2	4.8	5.1	8.1	5.9	4.8
Myrcene	4.3	4.3	3.7	5.9	2.4	2.5	5.3	3.4	3.0	3.1	6.5	8.1	5.0
*p*-Cymene	5.3	5.0	2.9	1.9	7.4	2.7	4.6	3.5	2.1	2.0	2.3	4.0	4.2
1-Methoxydecane	0.1	0.0	17.6	0.0	0.0	0.9	0.4	2.2	12.1	9.0	7.8	9.2	0.0
Serratol	2.2	0.1	0.5	5.9	0.2	0.9	0.2	1.1	0.3	0.3	0.4	0.6	0.8
Unident. guaiol	0.0	0.0	0.4	0.0	0.0	0.0	0.0	0.0	0.2	0.2	0.3	0.2	0.0
1-Methoxyoctane	0.0	0.0	5.5	0.0	0.0	0.3	trace	0.7	3.8	2.6	2.1	2.7	0.0
